# Supplementary material for: Identification of photoperiod-induced specific miRNAs in the adrenal glands of Sunite sheep (Ovis aries)
Source: Front Vet Sci. 2022 Jul 22;9:888207. doi: 10.3389/fvets.2022.888207 (PMC9354845; doi:10.3389/fvets.2022.888207)
Supplement: Supplementary file 1 [file Data_Sheet_1.ZIP › Supplementary file/miRDeep2 file.zip!/miRDeep2 file/Know_Structure/SPLP42A2/oar-mir-191.pdf]

miRBase precursor : oar-mir-191
Total read count : 108922
oar-miR-191 read count : 108643
remaining reads : 279

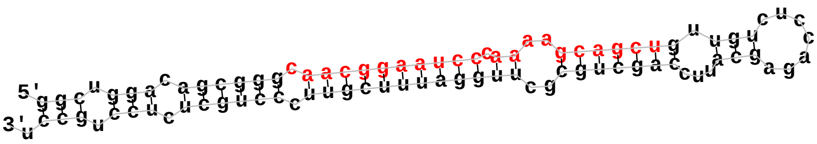

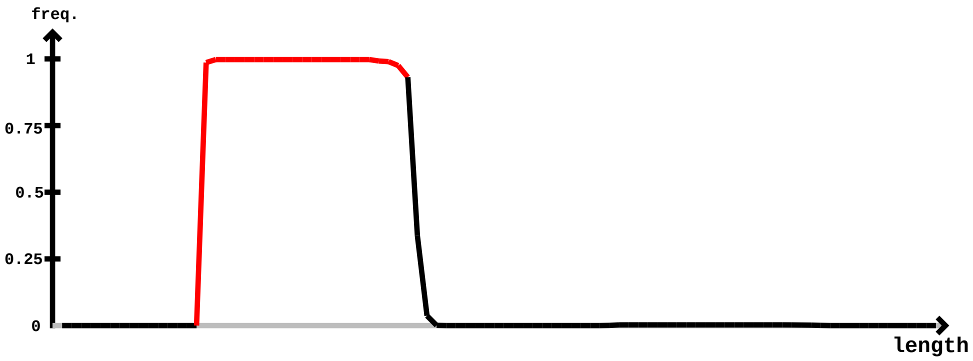


```
                 oar-miR-191
5'- ggcuggacagcgggcaacggaaucccaaaagcagcuguugucuccagagcauuccagcugcgcuuggauuucguucccugcucuccugccu  -3'    exp
    (((.(((.((((((.((((((((((.((..(((((((.(((.......)))...)))))))..))))))))))))).)))))).))).))).   reads     mm        sample
    ..............caacggaaucccaaaagc...........................................................   601       0         seq
    ..............caacggaaucccaaaagca..........................................................   237       0         seq
    ..............caacggaaucccaaaagcag.........................................................   1607      0         seq
    ..............caacggaaucccaaaagcagc........................................................   4727      0         seq
    ..............caacggaaucccaaaagcagcu.......................................................   64427     0         seq
    ..............caacggaaucccaaaagcagcug......................................................   32063     0         seq
    ..............caacggaaucccaaaagcagcugu.....................................................   3689      0         seq
    ..............caacggaaucccaaaagcagcuguu....................................................   70        0         seq
    ..............caacggaaucccaaaagcagcuguug...................................................   2         0         seq
    ...............aacggaaucccaaaagc...........................................................   1         0         seq
    ...............aacggaaucccaaaagcag.........................................................   6         0         seq
    ...............aacggaaucccaaaagcagc........................................................   22        0         seq
    ...............aacggaaucccaaaagcagcu.......................................................   345       0         seq
    ...............aacggaaucccaaaagcagcug......................................................   593       0         seq
    ...............aacggaaucccaaaagcagcugu.....................................................   244       0         seq
    ...............aacggaaucccaaaagcagcuguu....................................................   1         0         seq
    ...............aacggaaucccaaaagcagcuguugu..................................................   1         0         seq
    ................acggaaucccaaaagcagc........................................................   1         0         seq
    ................acggaaucccaaaagcagcu.......................................................   3         0         seq
    ................acggaaucccaaaagcagcug......................................................   3         0         seq
    .......................................................agcugcgcuuggauuucguu................   1         0         seq
    .......................................................agcugcgcuuggauuucguuc...............   2         0         seq
    ........................................................gcugcgcuuggauuucguu................   6         0         seq
    ........................................................gcugcgcuuggauuucguuc...............   18        0         seq
    ........................................................gcugcgcuuggauuucguucc..............   20        0         seq
    ........................................................gcugcgcuuggauuucguuccc.............   10        0         seq
    ........................................................gcugcgcuuggauuucguucccu............   3         0         seq
    .........................................................cugcgcuuggauuucgu.................   1         0         seq
    .........................................................cugcgcuuggauuucguu................   13        0         seq
    .........................................................cugcgcuuggauuucguuc...............   15        0         seq
    .........................................................cugcgcuuggauuucguucc..............   33        0         seq
    .........................................................cugcgcuuggauuucguuccc.............   97        0         seq
    .........................................................cugcgcuuggauuucguucccu............   52        0         seq
    .........................................................cugcgcuuggauuucguucccug...........   2         0         seq
    ..........................................................ugcgcuuggauuucguuc...............   1         0         seq
```

oar-miR-191

```
ggcuggacagcgggcaacggaaucccaaaagcagcuguugucuccagagcauuccagcugcgcuuggauuucguucccugcucuccugccu
..........................................................ugcgcuuggauuucguuccc.............     1     0     seq
..........................................................ugcgcuuggauuucguucccu............     4     0     seq
```
